# The challenges of gout flare reporting: mapping flares during a randomized controlled trial

**DOI:** 10.1186/s41927-019-0075-6

**Published:** 2019-07-09

**Authors:** Novell Teoh, Gregory D. Gamble, Anne Horne, William J. Taylor, Kate Palmano, Nicola Dalbeth

**Affiliations:** 1 Faculty of Medical and Health Sciences, University of Auckland, 85 Park Rd, Grafton, Auckland, New Zealand; 2 Department of Medicine, Faculty of Medical and Health Sciences, University of Auckland, 85 Park Rd, Grafton, Auckland, New Zealand; 3 Department of Medicine, University of Otago Wellington, Wellington, New Zealand; 4 Coromandel, New Zealand

## Abstract

**Background:**

Methods of gout flare reporting in research settings are inconsistent and poorly defined. The aim of this study was to describe patterns of gout flare and assess the concurrent validity of different methods of flare reporting in a gout clinical trial.

**Methods:**

Daily flare diary entries including self-report of flare and pain scale from a randomised controlled trial of 120 patients with gout were analysed. Detailed pain-by-time plots for each participant were inspected and analysed for different methods of flare reporting for both self-report and the classification tree (CART)-defined flare developed by Gaffo in 2012. Concurrent validity for different methods of flare reporting were analysed.

**Results:**

Although the single gout flare had a ‘typical’ average pattern (peak on day 1 and resolution over 14 days), individual pain-by-time plots showed wide variation in pain intensity, duration and frequency of flares. Over the four-month study period, there were 84/120 (70%) participants who experienced at least one self-reported flare that was not a ‘typical’ flare. The time to first self-reported flare correlated poorly with other measures of gout activity and other methods of flare reporting. The number of days with flare (either self-reported or Gaffo-defined) and the area under the pain-by-time curve correlated most strongly with other measures of disease severity.

**Conclusion:**

There is wide variation in the patterns of flare over time in individuals with gout, leading to challenges for flare reporting in clinical trials. Time-dependent reporting strategies such as number of days with flare or area under the pain-by-time curve correlate well with other measures of gout disease severity and may provide a more accurate measure of flare burden.

**Trial registration:**

Clinical trial number: ACTRN12609000479202, registered 17/06/2009.

**Electronic supplementary material:**

The online version of this article (10.1186/s41927-019-0075-6) contains supplementary material, which is available to authorized users.

## Background

Recurrent flares of acute inflammatory arthritis are the central clinical feature of gout [1]. Flares cause severe pain, disability and poor health-related quality of life in people with gout [2–4]. Despite the importance of flares for patients [5], methods of flare reporting in gout research are inconsistent and poorly defined.

There has been recent progress defining a gout flare for use in clinical research. Elements of a gout flare were identified through patient surveys [6]. In 2012, Gaffo et al. described a preliminary definition of gout flare for use in clinical research based on this initial work; two definitions were described which captured patient reported elements including pain scores and self-report of flare [7]. In 2018, these definitions were validated in a separate large, multinational cohort of patients with gout [8]. Based on data from the Study for Updated Gout Classification Criteria (SUGAR) study [9], the 2015 ACR/EULAR gout classification criteria included time-dependent elements of flare; time to maximal pain within 24 h, resolution of symptoms in ≤14 days, complete resolution (to baseline level) between symptomatic episodes [10, 11].

Although the presence of being in the state of flare and the time characteristics of a single flare have been defined, the optimal method of reporting flares over time is unclear. In clinical trials of urate-lowering therapy, flares are typically reported as the percentage of participants with at least one flare or mean number of flares over a specified time period [12, 13]. In studies of anti-inflammatory prophylaxis, additional methods have been used including time to first flare, severity of flares, and average duration of all flares [14–16]. The lack of standardisation of flare reporting makes comparison between different treatments difficult. Furthermore, categorisation of flare data may not capture the severity, duration or impact of flares. The aim of this study was to describe patterns of gout flare and assess the concurrent validity of different methods of flare reporting.

## Methods

Flare diary entries from a randomised controlled trial of patients with gout were analysed [17]. After a 1 month run-in period, study participants (*n* = 120) were randomized to one of three treatment arms for 3 months: skim milk powder enriched with glycomacropeptide (GMP) and G600 milk fat extract (G600) (*n* = 40), or one of two control groups; skim milk powder alone (*n* = 40) or lactose control (*n* = 40). All other gout medications including urate-lowering therapy and anti-inflammatory medications (non-steroidal anti-inflammatory drugs, colchicine or prednisone) for both prophylaxis and treatment of flares were prescribed according to the discretion of the patient’s usual doctor. Participants completed flare diary entries each day with recording of pain score (Likert scale 0–10) and self-report of flare for the month prior to randomisation and for the further 3 months following randomisation. The number of flares defined by self-report and by the 2012 Gaffo definition using the classification tree (CART) approach (pain score > 3 and self-report) were counted and reported as outcomes in the trial [7]. This study reported a greater improvement from baseline in number of gout flares (both by self-report and Gaffo CART-defined) over a three-month treatment period with the skim milk powder/GMP/G600 treatment. The trial was approved by the New Zealand Ministry of Health ethics committee (NTY/09/01/002) and all patients provided written informed consent. The trial was prospectively registered with the Australian New Zealand Clinical Trials Registry (ACTRN12609000479202, https://www.anzctr.org.au/Trial/Registration/TrialReview.aspx?id=83573). The study adheres to CONSORT guidelines.

In the current analysis, time series analysis anchored on the first self-reported or Gaffo CART-defined flare was used to model the average pain-by-time characteristics of a single flare. For the first flare pain-by-time models, the flare was defined as a contiguous period of time with non-zero pain scores bounded by 2 days of zero pain scores. Six diaries had no non-zero pain score data and two diaries had no sustained pain scores of zero; data from these participants were not included in the first flare pain-by-time model analysis, but were included in all subsequent analyses.

The patterns of flare over time were examined using the pain-by-time plots over the entire observation for all study participants. Cumulative probability plots were drawn to determine the variability of days with flare (adjusted for days of observation) and flare pain intensity for all study participants over the observation period. The time-course domain in the 2015 ACR/EULAR gout classification criteria was used to define the time elements of a ‘typical’ flare (time to maximal pain < 24 h, resolution of symptoms in ≤14 days, complete resolution (to baseline level) between symptomatic episodes) [11].

The association between different methods of flare reporting with other measures of disease activity over the observation period were analysed using Spearman correlations. For both self-reported flares and Gaffo CART-defined flares, the following methods of flare reporting were analysed: time to first flare adjusted for the duration of follow-up for each participant, number of flares, number of months with at least one flare, and number of days with flare. The area under the curve for the pain-by-time plot was also analysed. The measures of gout disease activity were analysed according to the area under the curve plots over the observation period, and included measures of joint inflammation (swollen joint count (/66), tender joint count (/68), and C-reactive protein), patient global assessment (Likert scale 0–5) and physician global assessment (Likert scale 0–5). All of the measures of gout activity were recorded at baseline and then monthly. The influence of baseline gout clinical characteristics were also analysed by correlation analysis. Data were analysed using SAS (v9.4 SAS Institute Inc. Cary, NC USA).

## Results

### Baseline characteristics

The flare diary entries for the 120 study participants had a mean (SD) follow-up period of 107 (25) days. Clinical features at baseline and over the observation period are shown in Table 1. Participants were predominantly middle aged men, with mean gout disease duration of 15 years. The mean (SD) number of self-reported flares in the 4 months prior to study entry was 4.5 (6.0). Approximately half of the participants were on allopurinol. There were 27% on colchicine, 13% on prednisone, and 50% on NSAIDs at study entry. Tophi were present in 36%. Mean (SD) serum urate at study entry was 0.42 (0.10) mmol/L.

**Table 1:** Clinical features of study population at baseline and over the 4 months observation period

Baseline	
Age, years, mean (SD)	56 (12)
Male, n (%)	107 (89%)
New Zealand European ethnicity, n (%)	74 (62%)
New Zealand Māori ethnicity, n (%)	20 (17%)
Pacific ethnicity, n (%)	16 (13%)
Asian ethnicity, n (%)	10 (8%)
Duration of gout, years, mean (SD)	15 (12)
No. of self-reported flares in preceding 4 months, mean (SD)	4.5 (6.0)
Allopurinol use, n (%)	64 (53%)
Colchicine use, n (%)	32 (27%)
Prednisone use, n (%)	16 (13%)
NSAID use, n (%)	60 (50%)
Presence of tophi, n (%)	43 (36%)
Serum urate, mmol/L, mean (SD)	0.42 (0.10)
Observation period	
Days to first self-reported flare, mean (SD)	13.1 (27.6)
Number of self-reported flares, mean (SD)	2.1 (2.0)
Number of months with ≥1 self-reported flare, mean (SD)	2.4 (1.3)
Days with self-reported flare, mean (SD)	21.1 (18.3)
Days to first Gaffo-CART defined, mean (SD)	41.3 (47.3)
Number of Gaffo-CART defined flares, mean (SD)	0.9 (1.0)
Number of months with ≥1 Gaffo-CART defined flare, mean (SD)	1.7 (1.3)
Days with Gaffo-CART defined flare, mean (SD)	7.9 (9.3)
Average pain score (range 0–10), mean (SD) ^a^	0.77 (1.74)
Average swollen joint count (/66), mean (SD)^b^	0.66 (1.57)
Average tender joint count (/68), mean (SD) ^b^	0.67 (1.24)
Average C-reactive protein, mg/L, mean (SD) ^b^	4.59 (7.47
Average physician global assessment (range 0–5), mean (SD) ^b^	1.46 (1.23)
Average patient global assessment (range 0–5), mean (SD) ^b^	1.46 (1.24)

^a^measured daily over the 4 months observation period, ^b^ measured monthly over the 4 months observation period

### Single flare model

**Fig. 1:**
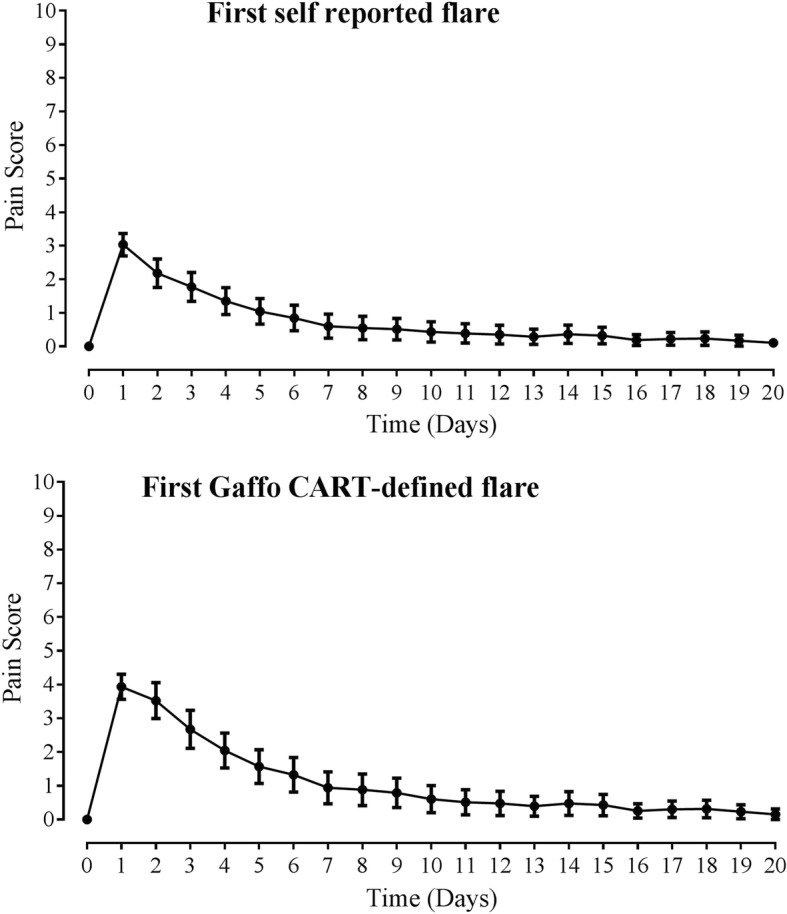
Average characteristics of a single flare; analysis of the first observed flare. Data are shown as mean (95% confidence interval)

There were 114 participants with at least one self-reported flare during the observation period. The mean (SD) number of self-reported flares over the observation period was 2.1 (2.0) and the mean number of flares fulfilling the Gaffo CART definition was 0.9 (1.0). The average pain-by-time plots for the first observed flare are shown in Fig. 1. The maximum pain score was higher for the Gaffo CART-defined flare, compared with self-reported flare (Additional file 1: Table S1). For these models of a single flare, the mean time to maximum pain was on the first day of the flare, and on average, the flare resolved after approximately 2 weeks.

### Flare patterns over the observation period

**Fig. 2:**
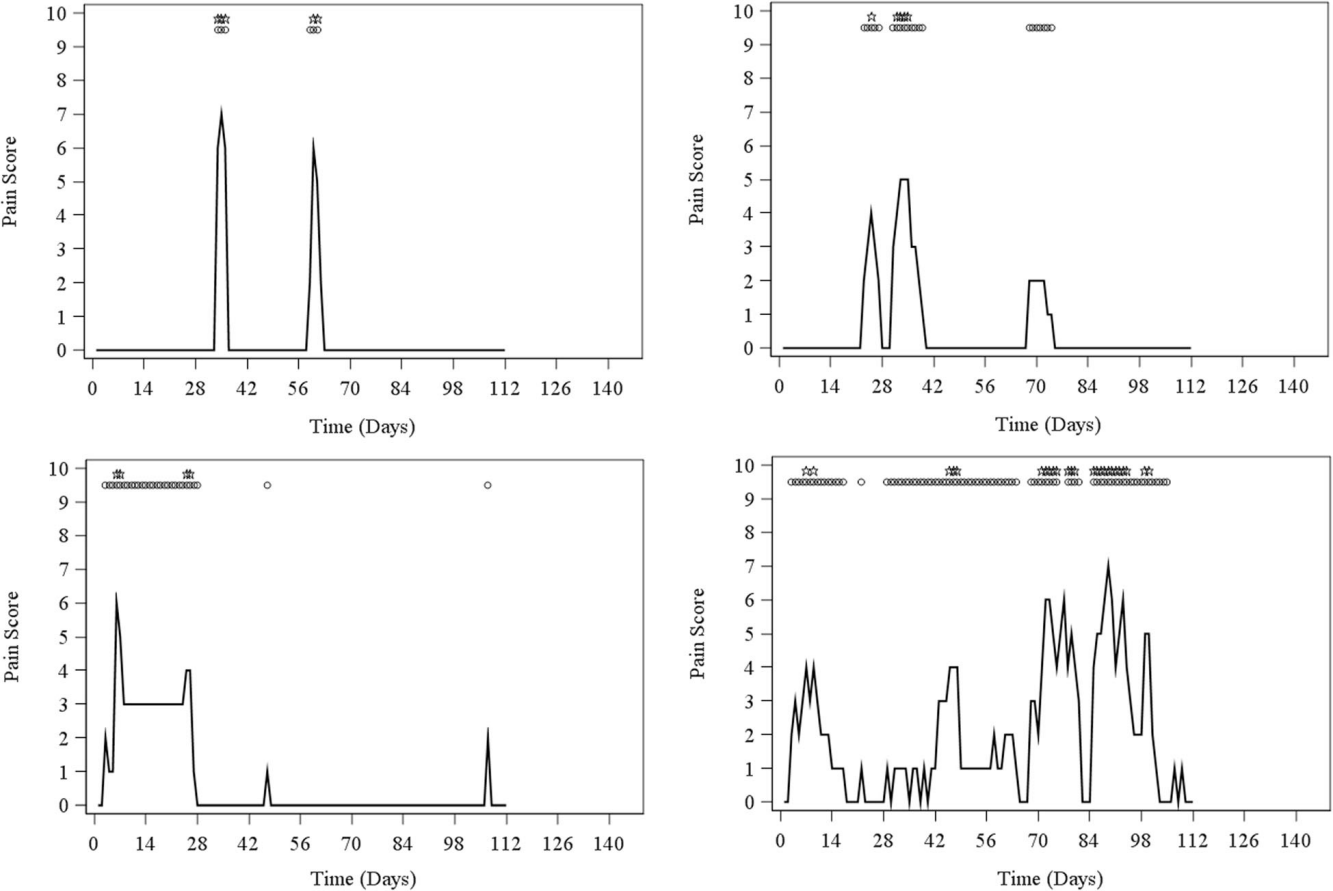
Examples of pain-by-time plots from four participants with more than one self-reported flare during the observation period demonstrating variations in patterns of flare. Dots represent days with self-reported flare. Stars represent days with Gaffo CART-defined flare

Although the single gout flare had an average ‘typical’ pattern according to the 2015 ACR/EULAR gout classification criteria, analysis of individual pain-by-time plots showed wide variation in the pain intensity, duration and frequency of flares. Figure 2 shows examples of the pain-by-time plots of four individuals with more than one self-reported flare during the observation period, demonstrating the wide range of flare patterns. Overall, there were 101/120 (84%) participants who experienced at least one self-reported ‘typical’ flare according to the 2015 ACR/EULAR gout classification criteria over the four-month study period, and 84/120 (70%) participants who experienced at least one self-reported flare that was not ‘typical’. There were 79/120 (66%) participants who experienced at least one Gaffo CART-defined flare ‘typical’ flare according to the 2015 ACR/EULAR gout classification criteria over the four-month study period, and 54/120 (45%) participants who experienced at least one Gaffo CART-defined flare that was not ‘typical’.

**Fig. 3:**
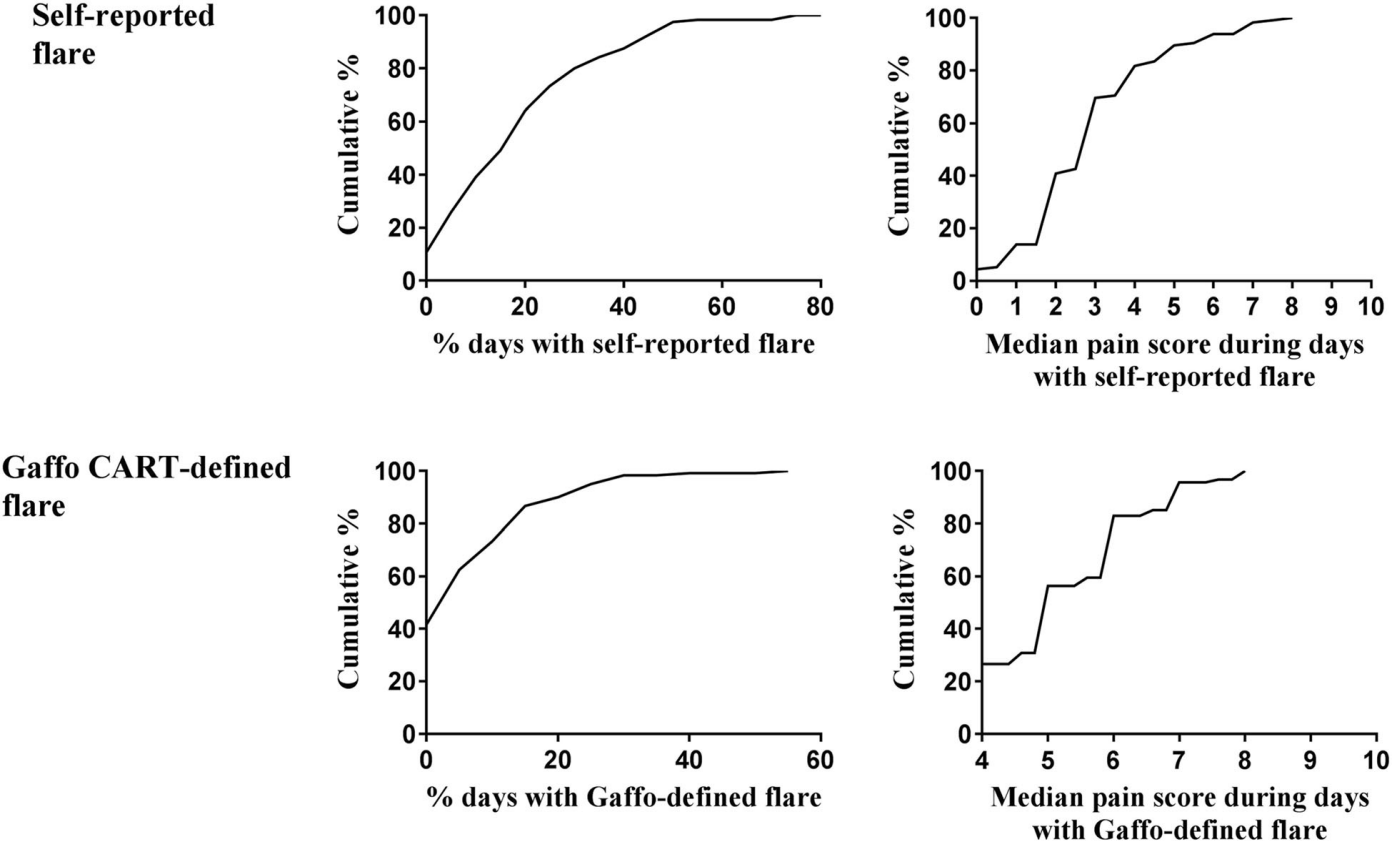
Cumulative probability plots showing the distribution of the percentage of days with flare and pain scores during flare for all study participants

Figure 3 confirms the variability, showing the distribution of these variables with cumulative probability plots for days with flare (adjusted for observation period) and pain scores during flare for all study participants. For all participants over the observation period, the median (range) percentage of days with self-reported flare was 18% (0–77%), and the median (range) percentage of days with Gaffo CART-defined flare was 4% (0–54%). For days with self-reported flares, the median (range) pain score was 3 (0–8), and for days with Gaffo CART-defined flare, the median (range) pain score was 5 (4–8).

### Concurrent validity with other measures of gout activity

Correlations of different methods of flare reporting with other measures of gout activity over the observation period were analysed (Table 2). The time to first self-reported flare correlated poorly with other measures of gout activity (Table 2) and other methods of flare reporting (Additional file 2: Table S2). In general, methods using the Gaffo CART-defined flare were more strongly correlated with other measures of gout activity compared with methods using self-reported flares, particularly with C-reactive protein. The number of days with flare (either self-reported or Gaffo CART-defined) and the area under the pain-by-time curve correlated most strongly with other measures of disease severity.

**Table 2:** Spearman correlations between methods of reporting flares and other measures of disease activity. ^a^adjusted for duration of follow-up for each participant. The area under the curve (AUC) for the measures of gout flare activity over the observation period was used in this analysis

		Self-reported flare				Gaffo CART-defined flare				AUC pain-by-time plot
		Time to first flare^a^	Number of flares	Number of months with ≥ 1 flare	Days with flare	Time to first flare^a^	Number of flares	Number of months with ≥ 1 flare	Days with flare	
Swollen joint count	r	−0.07	0.31	0.36	0.51	−0.20	0.37	0.42	0.41	0.50
	P	0.49	0.001	< 0.001	< 0.001	0.04	< 0.001	< 0.001	< 0.001	< 0.001
Tender joint count	r	−0.18	0.34	0.35	0.49	−0.20	0.29	0.35	0.33	0.44
	P	0.07	< 0.001	< 0.001	< 0.001	0.04	0.002	< 0.001	< 0.001	< 0.001
C-reactive protein	r	0.00	0.10	0.21	0.30	−0.31	0.24	0.28	0.32	0.34
	P	0.99	0.30	0.03	0.002	0.001	0.01	0.003	0.001	0.001
Patient global assessment	r	−0.19	0.44	0.45	0.62	−0.35	0.54	0.60	0.61	0.67
	P	0.04	< 0.001	< 0.001	< 0.001	< 0.001	< 0.001	< 0.001	< 0.001	< 0.001
Physician global assessment	r	−0.20	0.44	0.45	0.62	−0.35	0.54	0.60	0.60	0.67
	P	0.04	< 0.001	< 0.001	< 0.001	< 0.001	< 0.001	< 0.001	< 0.001	< 0.001

### Influence of baseline clinical characteristics on flares

In order to understand whether methods of flares reporting were influenced by baseline clinical characteristics, we analysed the correlations between methods of reporting with number of tophi, disease duration, number of gout flares in the prior 4 months before entering the study, and use of any anti-inflammatory medication use at the baseline visit (Additional file 3: Table S3). Methods of reporting reflected more frequent and severe flares in patients with longer disease duration and higher flare frequency at baseline. In particular longer disease duration correlated with higher number of flares, more months with at least one flare, and days with flare over the observation period. Participants with more flares in the prior 4 months and those taking anti-inflammatory medications also had shorter time to first flare during the observation period.

### Influence of baseline clinical characteristics on concurrent validity of time-dependent methods of flare reporting

The relationships between days with self reported flare and AUC pain-by-time plot with other measures of disease activity were analysed according to the gout clinical characteristics at baseline (Table 3). Overall, significant correlations with other measures of disease activity were observed irrespective of the tophus status, disease duration, or flare frequency. The associations with C-reactive protein were generally stronger in those with tophi, longer dissease duration, and more frequent flares. The observed correlations between joint counts and time-dependent measures of flare severity were not observed in those not taking anti-inflammatory medications at baseline, whereas a strong correlation was observed in those taking anti-inflammatory medications.

**Table 3:** Spearman correlations between time-dependent variables (days with self reported flare and AUC pain-by-time plot) with other measures of disease activity depending on gout clinical characteristics at baseline. The area under the curve (AUC) for the measures of gout flare activity over the observation period was used in this analysis

		No tophi, *n* = 77	Tophi, *n* = 43	Disease duration ≤ 12.5 years^a^, *n* = 63	Disease duration > 12.5 years, *n* = 57	Three or fewer flares in prior 4 months, *n* = 64	More than three flares in prior 4 months^b^, *n* = 56	No anti-inflammatory medications *n* = 38	Anti-inflammatory medications *n* = 82
*Days with self-reported flare*									
Swollen joint count	r	0.49	0.45	0.63	0.38	0.37	0.66	−0.23	0.73
	P	< 0.001	0.005	< 0.001	0.004	0.004	< 0.001	0.20	< 0.001
Tender joint count	r	0.42	0.65	0.56	0.43	0.46	0.52	−0.01	0.63
	P	< 0.001	< 0.001	< 0.001	0.001	< 0.001	< 0.001	0.98	< 0.001
C-reactive protein	r	0.19	0.38	0.16	0.44	0.13	0.48	0.28	0.27
	P	0.11	0.02	0.25	0.001	0.34	< 0.001	0.12	0.02
Patient global assessment	r	0.67	0.41	0.61	0.61	0.53	0.64	0.57	0.63
	P	< 0.001	0.01	< 0.001	< 0.001	< 0.001	< 0.001	< 0.001	< 0.001
Physician global assessment	r	0.66	0.42	0.62	0.61	0.52	0.65	0.59	0.63
	P	< 0.001	0.009	< 0.001	< 0.001	< 0.001	< 0.001	< 0.001	< 0.001
*AUC pain-by-time plot*									
Swollen joint count	r	0.48	0.47	0.68	0.30	0.41	0.55	−0.19	0.73
	P	< 0.001	0.004	< 0.001	0.03	0.001	< 0.001	0.28	< 0.001
Tender joint count	r	0.35	0.60	0.50	0.40	0.36	0.54	0.06	0.51
	P	0.002	< 0.001	< 0.001	0.002	0.005	< 0.001	0.73	< 0.001
C-reactive protein	r	0.23	0.54	0.26	0.42	0.21	0.52	0.20	0.37
	P	0.06	< 0.001	0.06	0.001	0.11	< 0.001	0.28	0.001
Patient global assessment	r	0.72	0.46	0.67	0.67	0.58	0.65	0.72	0.64
	P	< 0.001	0.004	< 0.001	< 0.001	< 0.001	< 0.001	< 0.001	< 0.001
Physician global assessment	r	0.72	0.45	0.67	0.65	0.58	0.65	0.72	0.64
	P	< 0.001	< 0.001	< 0.001	< 0.001	< 0.001	< 0.001	< 0.001	< 0.001

^a^Median disease duration was 12.5 years. ^b^Median number of flares in prior 4 months was 3

## Discussion

This analysis demonstrates that although the average gout flare has a characteristic pattern, there is wide variation in the patterns of flare over time in individuals with gout. The majority of participants in this study experienced at least one flare that did not conform to a typical pattern, as defined by the 2015 ACR/EULAR gout classification crtieria. This variation creates challenges for flare reporting in clinical trials. In particular, reporting the occurrence of a flare during a specified time period may not adequately represent the overall impact of flare, due to variation in duration, pain level, and intensity of inflammation.

The use of a more stringent flare definition which includes a pain domain in addition to self-report, such as the definitions of flare described by Gaffo, generally provides higher correlations with other measures of disease activity. For prolonged flares with fluctuating levels of pain, defining the start and stop time of the flare may be difficult, and could lead to inaccurate assessment of the number of flares. Time-dependent reporting strategies such as the number of days with flare or the area under the pain-by-time curve correlate well with other measures of gout disease severity and may provide a more accurate measure of severity of flare burden.

It is important to note that this analysis used data obtained from diaries that captured flare information on a daily basis. Recent large RCTs have recorded flare information using daily entries into electronic flare diaries [13, 16], and electronic capture of information about gout flares may allow easy capture of time-dependent flare information. This approach has the benefit of dynamic data capture, thereby avoiding issues of recall bias. However, a potential risk is incomplete recording of information in real-time, particularly in studies of long duration.

A central goal of gout management is complete suppression and prevention of flares. In a clinical trial setting, this may be feasible for medications with potent anti-inflammatory mechanisms of action for short term studies, but longer periods of treatment are usually required with urate-lowering agents to achieve this outcome. Methods of flare reporting that capture aspects of flare severity, such as intensity of symptoms, days with flare, or number of flares over a defined period may capture the experience of flare more comprehensively.

The clinical trial purposefully recruited people with frequent flares. Although the time-dependent methods of reporting had generally good concurrent validity in groups with different baseline gout characteristics, we did observe higher correlations with C-reactive protein in those with tophi, longer diseae duration and more frequent flares. Furthermore, correlations between swollen and tender joint counts were observed only in those taking anti-inflammatory medication at baseline. These findings suggest that the concurrent validity for the method of flare reporting may vary depending on the baseline characteristics. For studies of short duration, such as this clinical trial, this may be particularly relevant for patients with low disease activity who are not requiring anti-inflammatory medications at the time of recruitment into the study.

This analysis has some limitations. The site of flare was not recorded in the daily flare diaries, and therefore it is not possible to determine whether continuous reports of flare represent a prolonged flare in a single joint or new flares at different sites. The measures of measures of disease activity (C-reactive protein, joint counts, patient global asessments) were measured on a monthly basis, and it is possible that these measures did not capture all gout flares, particularly if they occurred between a study visit. Consistent with clinical practice, all patients had access to standard anti-inflammatory therapy for flare management, and it is possible that some methods of flare reporting were influenced by these therapies. However, other measures of disease activity such as joint counts and inflammatory markers would also be responsive to anti-inflammatory therapy. The study used the CART version of the Gaffo-defined flare, which has marginally lower accuracy than the 4-item version in a recent validation exercise (89% vs 92%) [8]. At the time of the study conduct, both versions were reported to have equivalent accuracy, and the Gaffo-CART version requires only two items, which was more feasible in a clinical trial setting. This work analysed daily flare diaries of 120 study participants over a 4 month period. Recent larger randomized controlled trials have recorded gout flare characteristics using daily flare diaries [16], including some studies for up to 1 year [13, 18, 19], and an individual participant data meta-analysis of these data would be of great interest to inform measurement of flare severity in future gout studies.

Many studies have reported that the experience of a gout flare is a major concern for people with gout [5, 20, 21]. Informed by semi-structured interviews with patients, and by patient partners with gout, pain, activity limitation, and flares were endorsed by OMERACT as mandatory domains for measurement in longterm clinical studies in gout [22]. Although instruments for both pain and activity limitation have been endorsed as valid instruments for longterm gout studies by OMERACT [23], no instrument for flare reporting has been endorsed [24]. Furthermore, methods of flare reporting are variable within clinical trials. Our analysis has described the measurement properties of different methods of flare reporting that are widely used in gout clinical trials, using data from an existing clinical study. A further important step in defining the most appropriate method of measurement is to understand from patients which aspects of the flare are most important. This future work is essential to guide meaningful flare reporting in future clinical trials.

## Conclusions

Patterns of flare over time vary widely in individuals with gout. Time-dependent reporting strategies such as the number of days with flare or the area under the pain-by-time curve correlate well with other measures of gout disease severity and may provide a more accurate and comprehensive assessment of flare burden.

## Additional files

Additional file 1: **Table S1.** Analysis of the first observed flare. (DOCX 14 kb)

Additional file 2: **Table S2.** Spearman correlations between different methods of flare reporting. *adjusted for duration of follow-up. *P* < 0.01 for all analyses. (DOCX 17 kb)

Additional file 3: **Table S3.** Correlations between methods of reporting flares and gout clinical characteristics at baseline. (DOCX 16 kb)

## Data Availability

All data generated or analysed during this study are included in this published article [and its Additional files 1, 2, and 3].

## Abbreviations

ACR: American College of Rheumatology
AUC: Area under the curve
CART: Classification and regression tree
EULAR: Euroepan League Against Rheumatism
G600: G600 milk fat extract
GEE: General estimating equation
GMP: Glycomacropepetide
OMERACT: Outcome Measures in Rheumatology
SD: Standard deviation
SUGAR: Study for Updated Gout Classification Criteria
