# Supplementary material for: The challenges of gout flare reporting: mapping flares during a randomized controlled trial
Source: BMC Rheumatol. 2019 Jul 9;3:27. doi: 10.1186/s41927-019-0075-6 (PMC6615178; doi:10.1186/s41927-019-0075-6)
Supplement: Supplementary file 1 — Table S1. Analysis of the first observed flare. (DOCX 14 kb) [file 41927_2019_75_MOESM1_ESM.docx]

**Additional file 1: Table S1.** Analysis of the first observed flare.

|  | **Self reported flare** | **Gaffo CART-defined flare** |
| --- | --- | --- |
| **Maximum pain score, median (IQR)** | 3 (2, 6) | 5 (4, 7) |
| **Time to maximum pain (days), median (IQR)** | 1 (1, 2) | 2 (1, 3) |
| **Time from maximum pain to resolution (days), median (IQR)** | 11 (7, 17) | 16 (10, 35) |
