# Supplementary material for: The challenges of gout flare reporting: mapping flares during a randomized controlled trial
Source: BMC Rheumatol. 2019 Jul 9;3:27. doi: 10.1186/s41927-019-0075-6 (PMC6615178; doi:10.1186/s41927-019-0075-6)
Supplement: Supplementary file 2 — Table S2. Spearman correlations between different methods of flare reporting. *adjusted for duration of follow-up. P < 0.01 for all analyses. (DOCX 17 kb) [file 41927_2019_75_MOESM2_ESM.docx]

**Additional file 2: Table S2.** Spearman correlations between different methods of flare reporting. *adjusted for duration of follow-up. P<0.01 for all analyses.

|  | | **Self-reported flare** | | | | **Gaffo CART-defined flares** | | | | **AUC pain-by-time plot** |
| --- | --- | --- | --- | --- | --- | --- | --- | --- | --- | --- |
|  |  | **Time to first flare*** | **Number of flares** | **Number of months with ≥1 flare** | **Days with flare** | **Time to first flare*** | **Number of flares** | **Number of months with ≥1 flare** | **Days with flare** |  |
| **Self-reported flare** | **Time to first flare*** | - | -0.32 | -0.57 | -0.48 | 0.51 | -0.27 | -0.40 | -0.32 | -0.40 |
|  | **Number of flares** | -0.32 | - | 0.60 | 0.66 | -0.27 | 0.62 | 0.48 | 0.43 | 0.54 |
|  | **Number of months with ≥1 flare** | -0.57 | 0.60 | - | 0.72 | -0.48 | 0.53 | 0.71 | 0.53 | 0.62 |
|  | **Days with flare** | -0.48 | 0.66 | 0.72 | - | -0.51 | 0.62 | 0.70 | 0.71 | 0.88 |
| **Gaffo CART-defined flares** | **Time to first flare*** | 0.51 | -0.27 | -0.48 | -0.51 | - | -0.67 | -0.69 | -0.74 | -0.66 |
|  | **Number of flares** | -0.27 | 0.62 | 0.53 | 0.62 | -0.67 | - | 0.87 | 0.86 | 0.76 |
|  | **Number of months with ≥1 flare** | -0.40 | 0.48 | 0.71 | 0.70 | -0.69 | 0.87 | - | 0.86 | 0.80 |
|  | **Days with flare** | -0.32 | 0.43 | 0.53 | 0.71 | -0.74 | 0.86 | 0.86 | - | 0.91 |
| **AUC pain-by-time plot** | | -0.40 | 0.54 | 0.62 | 0.88 | -0.66 | 0.76 | 0.80 | 0.91 | - |
