# Supplementary material for: The challenges of gout flare reporting: mapping flares during a randomized controlled trial
Source: BMC Rheumatol. 2019 Jul 9;3:27. doi: 10.1186/s41927-019-0075-6 (PMC6615178; doi:10.1186/s41927-019-0075-6)
Supplement: Supplementary file 3 — Table S3. Correlations between methods of reporting flares and gout clinical characteristics at baseline. (DOCX 16 kb) [file 41927_2019_75_MOESM3_ESM.docx]

**Additional file 3: Table S3.** Correlations between methods of reporting flares and gout clinical characteristics at baseline

|  |  | **Self-reported flare** | | | | **Gaffo CART-defined flare** | | | | **AUC pain-by-time plot** |
| --- | --- | --- | --- | --- | --- | --- | --- | --- | --- | --- |
|  |  | **Time to first flare*** | **Number of flares** | **Number of months with ≥1 flare** | **Days with flare** | **Time to first flare*** | **Number of flares** | **Number of months with ≥1 flare** | **Days with flare** |  |
| **Number of tophi** | r | 0.01 | 0.21 | 0.09 | 0.23 | -0.06 | 0.18 | 0.08 | 0.15 | 0.17 |
|  | P | 0.95 | 0.02 | 0.32 | 0.01 | 0.51 | 0.05 | 0.36 | 0.11 | 0.07 |
| **Disease duration** | r | -0.14 | 0.30 | 0.34 | 0.23 | -0.20 | 0.27 | 0.26 | 0.20 | 0.17 |
|  | P | 0.12 | 0.001 | <0.001 | 0.01 | 0.03 | 0.004 | 0.005 | 0.03 | 0.07 |
| **Number of gout flares in prior four months** | r | -0.25 | 0.22 | 0.18 | 0.25 | -0.20 | 0.22 | 0.19 | 0.26 | 0.27 |
|  | P | 0.005 | 0.02 | 0.05 | 0.005 | 0.03 | 0.02 | 0.04 | 0.005 | 0.003 |
| **Anti-inflammatory medication use** | r | -0.25 | 0.06 | 0.13 | 0.20 | -0.25 | 0.16 | 0.24 | 0.23 | 0.24 |
|  | P | 0.006 | 0.50 | 0.15 | 0.03 | 0.007 | 0.09 | 0.009 | 0.01 | 0.01 |
